# Inter-rater Reliability in Assessing Exercise Fidelity for the Injury Prevention Exercise Programme *Knee Control* in Youth Football Players

**DOI:** 10.1186/s40798-019-0209-9

**Published:** 2019-08-07

**Authors:** Gustav Ljunggren, Nirmala Kanthi Panagodage Perera, Martin Hägglund

**Affiliations:** 1 Department of Orthopaedics and Department of Medical and Health Sciences, Linköping University, Linköping, Sweden; 2 Division of Physiotherapy, Department of Medical and Health Sciences, Linköping University, Linköping, Sweden; 3 Botnar Research Centre, Nuffield Department of Orthopaedics, Rheumatology and Musculoskeletal Sciences, University of Oxford, Oxford, UK; 4 Centre for Sport, Exercise and Osteoarthritis Research Versus Arthritis, United Kingdom Latrobe Sports and Exercise Medicine Research Centre, College of Science, Health and Engineering, Latrobe University, Bundoora, VIC 3086 Australia

**Keywords:** Football, Soccer, Injury prevention programme, Neuromuscular training, Exercise fidelity, Knee injury, Youth sports

## Abstract

**Background:**

To receive maximum benefits from injury prevention exercise programmes (IPEP) such as *Knee Control*, players need to perform the exercises as prescribed. But, exercise fidelity in IPEPs is seldom evaluated. We developed a checklist to assess exercise fidelity in the *Knee Control* IPEP, and the primary aim was to evaluate its inter-rater reliability. The secondary aim was to study *Knee Control* exercise fidelity in youth football players and compare sex differences.

**Methods:**

This observational study included 11 teams with male and female youth players (11–18 years). On average, the players trained with the *Knee Control* IPEP for 7 weeks (SD 1.4, range 6–10 weeks). After the training period, two physiotherapists attended a team training session to observe players executing exercises and individually assessed their performance of these exercises as correct or incorrect based on standardised criteria set in the fidelity checklist. Agreement between observers was assessed using Cohen’s kappa coefficient.

**Results:**

The observers agreed on 144 out of 160 (90%) observations (Kappa = 0.80, substantial agreement). Both observers agreed on correct exercise performance for 69 out of 144 observations (exercise fidelity 48%). Exercise fidelity was higher in females (56%) than males (40%), but the difference was not statistically significant (*p* = 0.18).

**Conclusion:**

The *Knee Control* exercise fidelity checklist had high inter-rater reliability with substantial agreement. The exercise fidelity was low, which could hamper the preventive effects of an IPEP. Understanding the reasons for low exercise fidelity is important and more effort should focus on increasing exercise fidelity alongside the implementation of IPEPs.

**Electronic supplementary material:**

The online version of this article (10.1186/s40798-019-0209-9) contains supplementary material, which is available to authorized users.

## Key Points

- The exercise fidelity checklist developed for the *Knee Control* injury prevention exercise programme has high inter-rater reliability (substantial agreement, Kappa 0.80) and can be used to determine whether players perform *Knee Control* exercise correctly.
- The majority of players did not perform the *Knee Control* injury prevention exercise programme as prescribed, with only 48% of the observed exercises being performed according to the instructions.
- Exercise fidelity of the female football players was higher than for males; however, this finding needs to be confirmed in future studies.

## Background

Large-scale randomised controlled trials (RCT) have shown that injury prevention exercise programmes (IPEP) can substantially reduce sports injuries [1–5]. For example, the evidence-based IPEP *Knee Control* (*Knäkontroll®*) reduced by up to 90% severe acute knee injuries [6] including 64% of anterior cruciate ligament (ACL) injuries [2] in female youth football players. However, the demonstrated efficacy of the IPEPs might not translate into lasting real-world effects, as adherence to IPEPs deteriorates after implementation [7, 8]. Adherence to IPEPs, in terms of performing the exercises as prescribed, is essential to reduce injury risk. Players that perform the IPEP with high compliance obtain greater preventive benefits [6, 9]. Therefore, high intervention fidelity is likely crucial to increase the effectiveness of an IPEP [10–13].

In a nutshell, intervention fidelity refers to the extent to which an IPEP has been implemented as intended, in a comparable manner among all study participants [14]. Intervention fidelity of an IPEP includes both exercise fidelity (athletes performing the exercises according to instructions) and utilisation fidelity (IPEP delivered and exercises in the IPEP are executed with the prescribed number of sets and repetitions) [13]. Further, the extent of exercise fidelity can be dependent on the utilisation fidelity including how the IPEP was delivered, received and executed. Intervention fidelity is, therefore, a key methodologic requirement and integral to the internal validity of any prevention trial [15, 16]. Although RCT reporting guidelines such as the Consolidated Standards of Reporting Trials (CONSORT) statement [17] is widely endorsed, the quality of reporting aspects of intervention fidelity reporting remains poor. For example, a systematic review of sports injury preventions trials found only 12% of all included studies reported aspects of intervention adaptation and less than 1% reported on maintenance of the IPEPs [18]. Thus, the exercise fidelity of an IPEP is seldom evaluated alongside the RCT. In community-level Australian-rules football, only 67% of the players performed the exercises in an IPEP as prescribed [10]. To our knowledge, no other studies have evaluated exercise fidelity in an IPEP. This is especially a problematic issue for IPEP trials, because without in-depth information about the extent to which participants complied with the prescribed IPEP (exercise and utilisation fidelity of the intervention), findings are of lesser value [19]; evaluation of intervention fidelity can facilitate correct interpretation of results (positive or negative) and prevent incorrect conclusions of an intervention outcome [10].

In medical and non-medical fields, checklists are used as cognitive aids to guide users to accurately complete a given task. Using the same principle, a checklist can be used as a tool to outline criteria of accurate IPEP exercise execution. Therefore, it functions as a support resource that simplifies conceptualization and recall of information of correct performance of the IPEP exercises and can be used to evaluate the exercise and utilisation fidelity. Although *Knee Control* is a well-established IPEP, fidelity to this programme has not been evaluated. For this reason, an exercise fidelity checklist was developed to assess the fidelity of the *Knee Control* IPEP*.* Typically, studies rely on multiple data collectors, but due to the variability among individuals, the question of consistency (agreement) among observers collecting exercise fidelity data arise [20]. Therefore, it is important to assess the extent to which different observers give consistent estimates of the same behaviour. The inter-rater reliability of the *Knee Control* IPEP checklist assesses the external consistency of the checklist; typically, higher inter-rater reliability means consistency of the data collected by multiple collectors. Therefore, the main aim of this study was to evaluate the inter-rater reliability of the *Knee Control* exercise fidelity checklist. A secondary aim was to study *Knee Control* exercise fidelity in youth football players and compare sex differences.

## Materials and Methods

### The *Knee Control* IPEP

*Knee Control* is an evidence-based sports IPEP that is designed to prevent lower limb injuries in team ball sports such as football, floorball, handball and basketball. The *Knee Control* IPEP consists of six exercises: one-legged knee squat, pelvic lift, two-legged knee squat, the bench, the lunge and jump/landing technique [2]. All exercises have four variations with progressing difficulty and a partner exercise. Exercises are performed in three sets with 8 to 15 repetitions in each set (or 15–30 s for the bench) (see ref [2] for detailed information about exercises included in the *Knee Control* IPEP). Five minutes of low-intensity running is recommended before performing the *Knee Control* exercises. Therefore, the whole *Knee Control* IPEP takes approximately 15 min and should be performed two to three times per week before training sessions and the running-warm-up also before matches.

### Development of the *Knee Control* Exercise Fidelity Checklist

The *Knee Control* exercise fidelity checklist (Additional file 1) was developed by physiotherapists with extensive experience in instructing and researching the *Knee Control* IPEP. The IPEP instructions on how to perform each of the exercises were used as the foundation for the checklist to ensure consistency between the delivery of exercises and assessment of fidelity. The *Knee Control* exercise fidelity checklist was designed for on-field use so that the observer could assess the execution of the *Knee Control* exercises as *correct* or *incorrect* based on standardised criteria. The number of criteria varied between two and six for the different exercises. Face validity of the checklist was ensured because it was based on instructions outlined in the *Knee Control* IPEP, which are used to teach coaches and players how to perform the exercises correctly (Additional file 2). Before this study, two physiotherapists (GL and TK) pilot-tested the checklist by observing a group of players performing the *Knee Control* IPEP (data not included). No significant changes were made to the final checklist as a result.

### Participants

A convenience sample of football clubs with youth teams in a municipality in Sweden was contacted for inclusion. Eleven youth teams (six male and five female teams) from two clubs agreed to participate in this observational study.

### Implementation of the *Knee Control* IPEP

Two members of the research team visited each of the 11 teams during a team training session at the beginning of the season (April 2017) to deliver practical instructions about the *Knee Control* IPEP (i.e. implementation session). The 45–60-min-long implementation session was available to all coaches and players in each team. During the implementation session, each of the six exercises, exercise progressions and key performance techniques were demonstrated. Coaches were instructed to start with the first exercise level (easiest) for all players in the team, and then progress to more difficult levels of the exercises when they assessed that their players could perform the exercises correctly with good technique. As per programme instructions, coaches could individualise the progression for each player, but in reality, due to lack of resources, progressions were generally made for the whole team together. If a coach had progressed the exercise level (more difficult) for their team, they were encouraged to use lower-level exercises (easier) intermittently for more variation. Similarly, coaches could use the partner exercises to create more variation in the IPEP. Coaches received written instructions about the *Knee Control* IPEP and exercise execution, with explanatory text and pictures. After the initial implementation session, the research team had no further influence on the execution or exercise progressions of the *Knee Control* IPEP. The coaches were responsible for carrying out the *Knee Control* IPEP with their teams during the remainder of the spring season two times per week.

### Exercise Fidelity Assessment

At the end of the spring season in June 2017 (mean 7, SD 1.4 weeks after programme implementation, range 6–10 weeks), two physiotherapists (GL and TK) attended a team training session to observe player performance of exercises in the *Knee Control* IPEP during a regular warm-up. There was minimal interaction between the observers and the team during observation sessions. Before the start of the training session, the observation order of the players was randomised and written down, and each player was given an identifier (e.g. yellow shirt) to ensure that the two observers assessed the same player and exercise execution at the same time. Players were unaware of whether they were being observed during the exercises. The exercises performed were assessed as *correct* or *incorrect* based on the standardised criteria on the *Knee Control* exercise fidelity checklist (Additional file 1). A complete set (between 8 and 15 repetitions or 15 and 30 s for the bench) was independently observed for each exercise, and the performance of the full set was taken into consideration when deciding on the overall assessment. For example, if a player performed 12 repetitions of an exercise, of which 10 with good technique and 2 with technique flaws, the exercise observation was graded as *correct*.

The regional ethical review board in Linköping granted ethics approval for this study (Dnr: 2017/109-31) and consent was obtained from all players and their legal guardians. The study was performed in accordance with the standards of ethics outlined in the Declaration of Helsinki.

### Statistical Analysis

The sample size was estimated using a nomogram [15]. Based on the exercise fidelity observations from a previous study (67% correct exercises and 87% observer agreement) [10], the prevalence of correct exercise performance was set to 70%, and the minimal acceptable observer agreement was set to 80%. The minimum number of observations was set to 118 to enable detection of 90% observer agreement [21], which is 10% greater than the minimal acceptable level of agreement.

Data were analysed using SPSS ® 24.0 (IBM SPSS Statistics 2014). Cohen’s kappa coefficient was used to assess the agreement between observers (inter-rater agreement). Kappa values of ≤ 0.20, 0.21–0.40, 0.41–0.60, 0.61–0.80, and 0.81–1 correspond to slight, fair, moderate, substantial, and almost perfect agreement, respectively [22]. Exercise fidelity was defined as both observers scoring the exercise as being performed correctly and expressed as a proportion of the total number of exercise observations. A chi-squared test was used to evaluate sex differences in exercise fidelity. Statistical significance was set at *p* ≤ 0.05.

## Results

In total, 123 different players (58 males and 65 females) were observed. The mean age for the male players was 14.4 years (SD 2.5, range 12–18) and females 12.9 years (SD 1.2, range 11–14). According to the coach reports, on average, *Knee Control* IPEP was used 11.5 times (SD 2.9, range 7–17) from implementation to the follow-up observation with a mean use of 1.6 times/week (SD 0.3, range 1–2).

### Inter-rater Reliability

A total of 160 exercises performed by the 123 players (37 players were observed during two different exercises) were assessed using the *Knee Control* exercise fidelity checklist. Of the 160 observations, both observers agreed on 144 observations (90% agreement) (Table 1). The Kappa coefficient was 0.80 (95% CI 0.71–0.89), which is considered a substantial agreement. Inter-rater agreement was similar for all six individual exercises in the *Knee Control* IPEP (Table 2).

**Table 1:** Observer agreement of the fidelity of 160 exercises observed in the *Knee Control* injury prevention exercise programme

		Observer 2		Total (*n*)
		Correct (*n*)	Incorrect (*n*)	
Observer 1	Correct (*n*)	69	10	79
	Incorrect (*n*)	6	75	81
	Total (*n*)	75	85	160

**Table 2:** Observation agreement for the six individual exercises in the *Knee Control* injury prevention exercise programme

	Observations (*n*)	Observations agreed (*n*)	Observation agreement (%)
One legged knee squat	45	39	87
Pelvic lift	20	19	95
Two legged knee squat	23	20	87
The bench	25	24	96
The lunge	22	21	95
Jump/landing	25	21	84

### Exercise Fidelity of the *Knee Control* IPEP

Of the 144 observations where both observers agreed, 48% (n = 69) of the exercises were performed correctly. Of the individual exercises, pelvic lift had the highest fidelity whilst jump/landing, one and two legged squats had the lowest fidelity (Table 3). The most common technique flaw in the latter exercises was poor knee-over-foot alignment (Additional file 3). Exercise fidelity was greater in females with 56% (40/72) of the observed exercises being performed correctly compared to 40% (29/72) in male players, although not statistically significant (*p* = 0.18).

**Table 3:** Distribution of *Knee Control* exercise observations assessed as correct

	Observations agreed total	Agreed correct total	Agreed correct boys	Agreed correct girls
One legged knee squat	39	15 (38%)	6 (27%)	9 (53%)
Pelvic lift	19	16 (84%)	7 (78%)	9 (90%)
Two legged knee squat	20	6 (30%)	4 (33%)	2 (25%)
The bench	24	13 (54%)	4 (40%)	9 (64%)
The lunge	21	13 (62%)	5 (56%)	8 (67%)
Jump/landing	21	6 (29%)	3 (30%)	3 (25%)

## Discussion

The main finding of this study was that the exercise fidelity checklist developed for the *Knee Control* IPEP showed substantial inter-rater reliability (Kappa 0.80). Another important finding was that the observed exercise fidelity was low among youth football players, with less than half of the exercises performed according to the instructions.

### The *Knee Control* Exercise Fidelity Checklist

Inter-rater agreement for the *Knee Control* exercise fidelity checklist (which was developed based on the exercise instructions) was high with a substantial agreement between the two observers. The high reliability means that the external consistency of the checklist is high, and this checklist can be used in future studies that assess exercise fidelity in the *Knee Control* IPEP. It should be noted that the checklist was used by two physiotherapists who were familiar with the *Knee Control* IPEP. Thus, findings may vary among those who are unfamiliar or less skilled in movement observations, such as coaches or physiotherapy students. This limits the wide implementation of the *Knee Control* exercise fidelity checklist without further support.

The inter-rater agreement was slightly lower for the exercises one- and two-legged knee squats and jump/landing (84–87%) compared with the other exercises in the IPEP (95–96%). This could be due to the rapid pace of the exercise execution and that the raters had a smaller window of opportunity to observe and assess these exercises. Another factor might be that the criteria used to assess these exercises might be inexplicit and broader and therefore more difficult to evaluate.

Based on the positive results of the initial RCT [2], nationwide dissemination of the *Knee Control* programme was initiated in 2010 by the Swedish Football Association, including the incorporation of *Knee Control* IPEP into the Swedish football-coach education curriculum [23]. Implementing the *Knee Control* checklist among coaches as a means to monitor their own players’ exercise fidelity might, in turn, increase the overall exercise fidelity among players. Also, coaches can use the checklist to identify exercises with low fidelity and focus on developing player skills/capacities to achieve high exercise fidelity. However, more support and resources should be provided to help the coaches to upskill and become confident about using the exercise fidelity checklist.

### Exercise Fidelity of the *Knee Control* IPEP

Exercise fidelity was low, with less than 50% of the observed exercises being performed as prescribed. This is of concern as low exercise fidelity may reduce the preventive effectiveness of an IPEP such as *Knee Control*. For example, in an RCT, a reduction in ACL injury rate by 64% [2] and up to 90% reduction of severe acute knee injuries was reported in players who were highly compliant to the *Knee Control* IPEP [6]. Adherence remains a challenge [6] and, as evinced by our study, exercise fidelity was low among youth players. Therefore, in the real-world sports setting, the effectiveness of the *Knee Control* IPEP could be severely reduced. Evidence from insurance data shows that when comparing the 5-year periods before and after the nationwide implementation of the *Knee Control* IPEP in Sweden, there has been a decrease in the incidence of cruciate ligament injuries by 13% in female players [23]. This reduction corresponds to approximately 100 less cruciate ligament injuries in Swedish football annually, but the effect is still considerably lower than the 64% reduction observed in the RCT [2]. Further support and follow-up with coaches (e.g. how to help players correct exercise performance, cues to instruct players) should be considered for implementation of IPEPs to achieve maximal benefits of these programmes.

We observed a sex difference in exercise fidelity, as female players demonstrated higher exercise fidelity than their male counterparts. A possible explanation might be that the preventative effects of *Knee Control* IPEP in girls’ football gained great media attention, which might have encouraged more coaches of female players to use *Knee Control* IPEP regularly in their training. Further, these coaches and female players might be more aware of the high risk of knee injuries in women and the effectiveness of *Knee Control* IPEP because of the heightened attention from media and football associations. Therefore, it is possible that *Knee Control* IPEP is well received by coaches and female players and they have more vested interests in performing the exercises correctly to achieve positive benefits. However, this finding needs to be confirmed in future studies. Furthermore, the age difference between boys and girls in our study, as well as the relatively small sample, means the observed sex-related difference should be viewed with caution.

## Limitations

In addition to the limitations discussed above, the following should also be considered. At times, the observers may not have had an optimal view to observe, for instance, the knee-over-foot alignment. Also, as discussed above, some of the criteria for assessing the exercise were broader and thus open for interpretation. However, both observers were familiar with the *Knee Control* IPEP, and they were also involved in pilot-testing the checklist.

The convenience sampling and small sample size may impact the external validity of our findings related to exercise fidelity and observed sex differences, even though we have no obvious reason to believe that this cohort of teams was systematically different from other teams in the same leagues. Further, we believe the team recruitment to be sufficient for the main aim of assessing the inter-rater reliability of the fidelity checklist. It is a limitation that the players and the coaches were aware that the physiotherapists were there to observe their training session. It is possible that the players, therefore, performed the exercises to a higher standard and that coaches may have instructed their players to perform the exercises more conscientiously. Therefore, it is possible that our study may overestimate exercise fidelity. On the other hand, the effects of being observed may also lead to poorer exercise execution, e.g. due to heightened tension or nervousness. The physiotherapists kept the communication and interaction with the team to a minimum, and the coaches and players were not aware which exercises and players were being assessed, in order to reduce the impact of observers being on the field.

Finally, as discussed above, coach self-efficacy is an important aspect of intervention adherence; yet, we did not assess the delivery of the IPEP and as such assume that the *Knee Control* IPEP was delivered and performed as intended. We only assessed the exercise fidelity of the players in one session; therefore, it is unknown to what extent the coaches were delivering the *Knee Control* IPEP according to the instructions.

## Conclusions

The checklist used to assess exercise fidelity of *Knee Control* IPEP had high inter-rater reliability, with substantial agreement. The exercises observed were generally performed with low fidelity. This is of concern as low exercise fidelity could hamper the preventive effects of a potent IPEP such as *Knee Control*. Therefore, it is important to understand the reasons for low exercise fidelity. More effort should also focus on increasing exercise fidelity alongside the implementation of IPEP.

## Additional files

Additional file 1: Checklist for exercise fidelity. (DOCX 16 kb)

Additional file 2: Criteria for correct performance. (DOCX 15 kb)

Additional file 3: Exercise fidelity for the observer agreed *Knee Control* exercises. (DOCX 19 kb)

## Data Availability

All data generated or analysed during this study are included in this published article [and its supplementary information files].

## Abbreviations

ACL: Anterior Cruciate Ligament
IPEP: Injury Prevention Exercise Programmes
RCT: Randomised Controlled Trials
