# Supplementary material for: Inter-rater Reliability in Assessing Exercise Fidelity for the Injury Prevention Exercise Programme Knee Control in Youth Football Players
Source: Sports Med Open. 2019 Aug 7;5:35. doi: 10.1186/s40798-019-0209-9 (PMC6686029; doi:10.1186/s40798-019-0209-9)
Supplement: Supplementary file 1 — Checklist for exercise fidelity. (DOCX 16 kb) [file 40798_2019_209_MOESM1_ESM.docx]

| **1. One legged knee squat** | **Correct** | **Incorrect** | **Criteria not met** |
| --- | --- | --- | --- |
| Overall assessment |  |  | - Knee over foot alignment - Trunk control - Stable hip - Whole foot contact ground - Foot pointing forward |
| Exercise level: A B C D Pair exercise  Number of repetitions: | | |  |
| Player ID: | | |  |
| **2. Pelvic lift** | **Correct** | **Incorrect** | **Criteria not met** |
| Overall assessment |  |  | - Trunk control - Hip extension |
| Exercise level: A B C D Pair exercise  Number of Repetitions: | | |  |
| Player ID: | | |  |
| **3. Two legged knee squat** | **Correct** | **Incorrect** | **Criteria not met** |
| Overall assessment |  |  | - Knee over foot alignment - Upright position trunk - Knee flexion to 90° - Feet pointing forward |
| Exercise level: A B C D Pair exercise  Number of Repetitions: | | |  |
| Player ID: | | |  |
| **4. The bench** | **Correct** | **Incorrect** | **Criteria not met** |
| Overall assessment |  |  | - Elbows beneath shoulders - Upper body & trunk straight - For side-bench: one elbow beneath shoulder |
| Exercise level: A B C D Pair exercise  Number of Repetitions: | | |  |
| Player ID: | | |  |
| **5. The lunge** | **Correct** | **Incorrect** | **Criteria not met** |
| Overall assessment |  |  | - Knee over foot alignment - Trunk control - Stable hip - Forward knee flexed to 90° - Foot pointing forward |
| Exercise level: A B C D Pair exercise  Number of Repetitions: | | |  |
| Player ID: | | |  |
| **6. Jump & landing** | **Correct** | **Incorrect** | **Criteria not met** |
| Overall assessment |  |  | - Knee over foot alignment - Trunk control - Knee control - Controlled jump & landing - Soft landing - Foot pointing forward at landing |
| Exercise level: A B C D Pair exercise  Number of Repetitions: | | |  |
| Player ID: | | |  |

Electronic Supplementary Material Appendix S1 Checklist for exercise fidelity
