# Supplementary material for: Inter-rater Reliability in Assessing Exercise Fidelity for the Injury Prevention Exercise Programme Knee Control in Youth Football Players
Source: Sports Med Open. 2019 Aug 7;5:35. doi: 10.1186/s40798-019-0209-9 (PMC6686029; doi:10.1186/s40798-019-0209-9)
Supplement: Supplementary file 2 — Criteria for correct performance. (DOCX 15 kb) [file 40798_2019_209_MOESM2_ESM.docx]

Electronic Supplementary Material Appendix S2 criteria for correct performance

**1. One legged knee squat**

- During one legged knee squat, knee motion forward in the sagittal plane, alignment with foot
- Trunk control maintained with minimum lateral movement allowed
- Stable hip with horizontal pelvic position
- The sole of the foot in contact with the ground
- Foot placed in the sagittal plane pointing forward

**2. Pelvic lift**

- Trunk control with minimum wobbling allowed in the frontal plane
- Full hip extension to 0° (neutral position)

**3. Two legged knee squat**

- Knee over foot alignment during the squat
- The trunk should be upright
- Knee flexion to 90°
- Feet pointing forward and the sole of the feet in contact with the ground

**4. The bench**

- Elbows placed beneath the shoulders with 90° flexion of the shoulders and elbows
- Upper body and trunk kept straight, no hip flexion/extension

Side bench:

- Elbow under shoulder with 90° shoulder abduction and 90° elbow flexion
- No hip abduction/adduction

**5. The lunge**

- Knee over foot alignment
- Trunk control maintained with minimum lateral movement allowed
- Stable hip with horizontal pelvic position
- Forward knee flexed to 90°
- Foot placed in the sagittal plane pointing forward

**6. Jump & landing**

- Knee over foot alignment
- Trunk control maintained with minimal motion allowed
- Knee control maintained with only minimal wobble sideways allowed
- Controlled and soft landing
- Foot placed in sagittal plane pointing forward at landing
