# Supplementary material for: Inter-rater Reliability in Assessing Exercise Fidelity for the Injury Prevention Exercise Programme Knee Control in Youth Football Players
Source: Sports Med Open. 2019 Aug 7;5:35. doi: 10.1186/s40798-019-0209-9 (PMC6686029; doi:10.1186/s40798-019-0209-9)
Supplement: Supplementary file 3 — Exercise fidelity for the observer agreed Knee Control exercises. (DOCX 19 kb) [file 40798_2019_209_MOESM3_ESM.docx]

Electronic Supplementary Material Appendix S3. Exercise fidelity for the observer agreed *Knee Control* exercises

| **Knee Control exercise** | **Performance criteria for the exercise** | **Fidelity of the criterion** | | **Fidelity of the whole exercise** | |
| --- | --- | --- | --- | --- | --- |
|  |  | **Correct**  **n (%)** | **Incorrect**  **n (%)** | **Correct**  **n (%)** | **Incorrect**  **n (%)** |
|  |  |  |  |  |  |
| **1. One legged knee squat** | Knee-over-foot alignment‎ | 20 (51) | 19 (49) | 15 (38) | 24 (62) |
|  | Trunk control | 31 (79) | 8 (21) |  |  |
|  | Stable hip | 35 (90) | 4 (10) |  |  |
|  | Whole foot contact with ground | 36 (92) | 3 (8) |  |  |
|  | Foot‎ pointing forward | 38 (95) | 2 (5) |  |  |
|  |  |  |  |  |  |
| **2. Pelvic lift** | Trunk control | 19 (100) | 0 (0) | 16 (84) | 3 (16) |
|  | Hip extension | 16 (84) | 3 (16) |  |  |
|  |  |  |  |  |  |
| **3. Two legged knee squat** | Knee-over-foot alignment | 12 (60) | 8 (40) | 6 (30) | 14 (70) |
|  | Upright position trunk | 14 (70) | 6 (30) |  |  |
|  | Flexion of hip, knee and foot | 14 (70) | 6 (30) |  |  |
|  | Knee flexion to 90° | 11 (55) | 9 (45) |  |  |
|  | Feet pointing forward | 19 (95) | 1 (5) |  |  |
|  |  |  |  |  |  |
| **4. The bench** | Elbows beneath shoulders | 23 (96) | 1 (4) | 13 (54) | 11 (46) |
|  | Upper body & trunk straight | 13 (54) | 11 (46) |  |  |
|  | For side-bench: one elbow beneath shoulder | - | - |  |  |
|  |  |  |  |  |  |
| **5. Lunges** | Knee-over-foot‎ alignment | 16 (76) | 5 (24) | 13 (62) | 8 (28) |
|  | Trunk control | 19 (90) | 2 (10) |  |  |
|  | Stable hip | 19 (90) | 2 (10) |  |  |
|  | Forward knee flexed to 90° | 18 (86) | 3 (14) |  |  |
|  | Foot‎ pointing forward | 20 (95) | 1 (5) |  |  |
|  |  |  |  |  |  |
| **6. Jump and landing** | Knee-over-foot alignment | 13 (62) | 8 (38) | 6 (29) | 15 (71) |
|  | Trunk control | 18 (86) | 3 (14) |  |  |
|  | Knee control | 13 (62) | 8 (38) |  |  |
|  | Controlled jump & landing | 15 (71) | 6 (29) |  |  |
|  | Soft landing | 13 (62) | 8 (38) |  |  |
|  | Foot pointing forward at landing | 20 (95) | 1 (5) |  |  |
